# A Validated Ontology for Metareasoning in Intelligent Systems

**DOI:** 10.3390/jintelligence10040113

**Published:** 2022-11-24

**Authors:** Manuel F. Caro, Michael T. Cox, Raúl E. Toscano-Miranda

**Affiliations:** 1 Education, Technology & Language (EduTLan Research Group), Department of Educational Informatics, University of Córdoba, Carrera 6 No. 77-305, Montería 230002, Córdoba, Colombia; 2 Education, Department of Computer Science & Engineering, College of Engineering and Computer Science, Wright State University, Dayton, OH 45324, USA

**Keywords:** metareasoning ontology, intelligent systems, metareasoning problem, ontology validation, heterogeneity problem

## Abstract

Metareasoning suffers from the heterogeneity problem, in which different researchers build diverse metareasoning models for intelligent systems with comparable functionality but differing contexts, ambiguous terminology, and occasionally contradicting features and descriptions. This article presents an ontology-driven knowledge representation for metareasoning in intelligent systems. The proposed ontology, called IM-Onto, provides a visual means of sharing a common understanding of the structure and relationships between terms and concepts. A rigorous research method was followed to ensure that the two main requirements of the ontology (integrity based on relevant knowledge and acceptance by researchers and practitioners) were met. The high accuracy rate indicates that most of the knowledge elements in the ontology are useful information for the integration of multiple types of metareasoning problems in intelligent systems.

## 1. Introduction

Metareasoning refers to the processes that monitor the progress of our reasoning and problem-solving activities and regulate the time and effort devoted to them (Ackerman and Thompson 2017; Horvitz 1987; Russell and Wefald 1991). Metareasoning is often defined as reasoning about reasoning (e.g., Hayes-Roth et al. 1983; Kuokka 1991), which implies intelligent decisions about how to think (Griffiths et al. 2019). In research on artificial intelligence, metareasoning plays a central role in the definition and design of rational agents that can operate on performance-limited hardware and interact with their environment in real time (Conitzer and Sandholm 2003; Dannenhauer et al. 2014; Svegliato and Zilberstein 2018). In the cognitive systems research community, metareasoning (or computational metacognition) is key to modeling high-level decision making, self-explanation and introspection (Cox 2011; Dannenhauer et al. 2018). Metareasoning also enables intelligent agents to optimize their own decision-making process to produce effective action in a timely manner (Svegliato et al. 2021).

However, metareasoning suffers from the heterogeneity problem, where differing metareasoning models with similar functionalities are being developed for intelligent agents in heterogeneous environments by different researchers. These models have conflicting features, descriptions, qualities, algorithms, and non-standard conventions because they come from different areas of knowledge, such as cognitive science (Ackerman and Thompson 2017), psychology (Dunlosky and Bjork 2008), education (Caro et al. 2014), computer science (Borghetti and Gini 2008), engineering (Caleiro et al. 2005), and AI (Conitzer and Sandholm 2003; Cox and Raja 2011; Svegliato and Zilberstein 2018). Due to these types of heterogeneities and the increasing application domains (e.g., online planning, anytime algorithms, brittleness problem of AI systems, introspective systems and long-duration missions), the research community now needs a common understanding of the terms and concepts related to metareasoning in intelligent systems. In this sense, there is a requirement to create a standard or global ontology that provides a common knowledge representation of the metareasoning domains.

Therefore, the main objective of this paper is to present an ontology that allows specifying a common language and a conceptualization of metareasoning in the domain of AI, which can be used to represent different metareasoning problems in intelligent systems. An ontology is defined as a formal specification of a shared conceptualization (Gruber 1995). Furthermore, ontologies allow the reuse of domain knowledge, thus making domain assumptions explicit and helping us to clarify any ambiguities (Horridge et al. 2019; Noy and McGuinness 2001).

Several studies have constructed ontologies for describing various aspects of metareasoning. Unfortunately, no comprehensive set of standardized features exists for describing the metareasoning domain. Therefore, each study has developed partial ontologies to address specific problems such as failures in AI systems (Schmill et al. 2007), the metacognitive cycle (Schmill et al. 2011) and meta-level control (Madera-Doval 2019). A general ontology for broadly describing the metareasoning domain and detailed ontologies for each metareasoning problem is still missing in the literature.

The main contributions of this work are:

- The Integrated Metareasoning Ontology, an ontology for the representation of different metareasoning problems in intelligent systems. We describe a framework for the use of the formally defined semantics of the classes, the individuals, and the properties of the ontology to construct the knowledge representation structure necessary to monitor and control the reasoning processes in intelligent agents. The proposed ontology also reuses existing ontologies that are used for partial modelling of some aspects of the metareasoning domain.

The remaining sections of the paper are as follows. Section 2 sketches the research methodology used in our work, and Section 3 details the development and validation of the ontology using this approach. It also describes a case study that applies the ontology to the problem of allocating student teams in internship programs. Section 4 then provides a discussion of the results; the summary of the study’s key findings and its limitations is included in Section 5.

## 2. Materials and Methods

Approaches to ontology development have been evolving in recent years. Authors have proposed updated methodologies based on the review and identification of the limitations of existing ontology development methods.

The methodology employed for ontology development is a hybrid framework based on DSR (Hevner et al. 2004; Peffers et al. 2012), which leverages the “methontology” approach (Baccigalupo and Plaza 2007), the 7-step methodology for ontology development (Noy and McGuinness 2001) and the “Uschold and Gruninger” ontology building approach (Uschold and Gruninger 1996). The Uschold and Gruninger approach provides detailed information for delineating the purpose and scope, ontology formalization, evaluation, and documentation. On the other hand, the methontology approach provides a more nuanced approach toward knowledge acquisition, conceptualization, and implementation.

**Figure 1:**
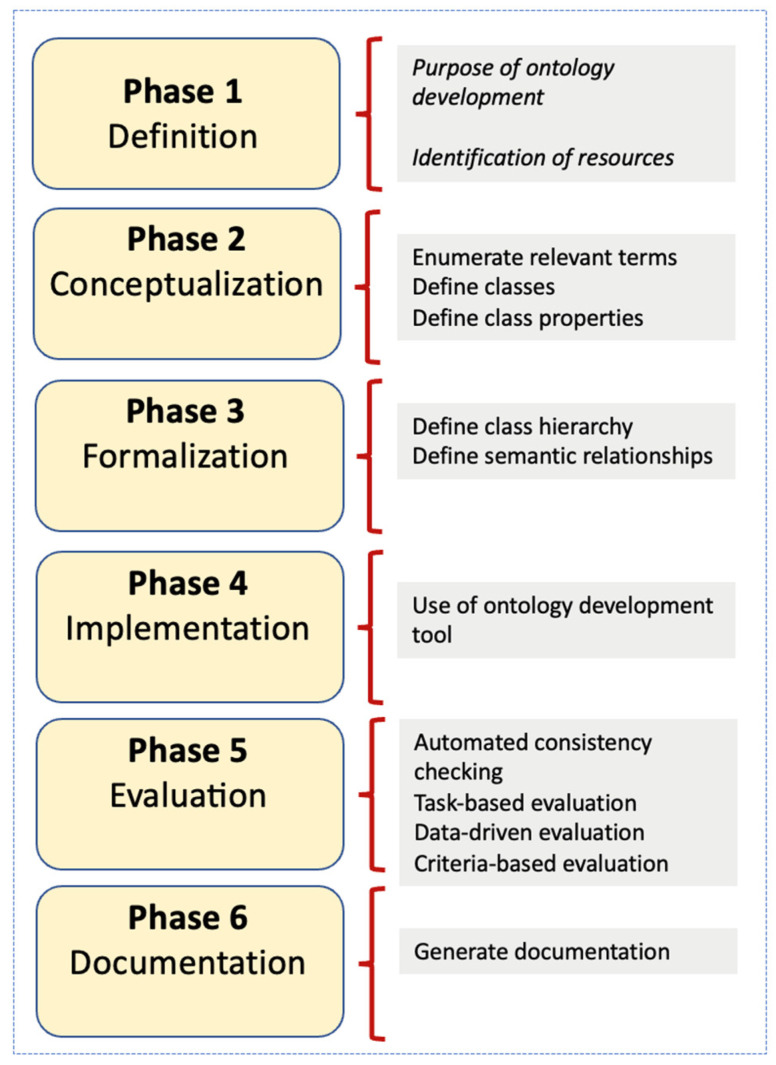
Research method. Source: Elaborated by the authors based on (Badr et al. 2013).

These phases have been identified in other studies, such as Badr et al. (2013). Figure 1 depicts this step-by-step research framework for developing the ontology and implementing it pragmatically in a case study.

Based on this framework, it was first important to define the scope and purpose of the ontology and to identify key resources from which the ontology is derived (Phase 1). This was executed by enumerating and answering a set of ontology specification questions and by listing specific functional roles for the ontology. This phase was followed by knowledge capture and the abstraction of relevant terms and their relationships in the domain from the resources (Phase 2). The formalization process is then performed to produce meaningful models based on semantic relationships (Phase 3). This phase identifies the hierarchies of subclasses as well as the existing semantic relationships between the different classes. Beyond this stage, the ontology was formally coded using RDF/OWL (Resource Description Framework/Web Ontology Language) (Van Assem et al. 2006) to make it computer-interpretable (Phase 4). The coded ontology underwent internal logical checks and was subsequently implemented in the case study. Further validation of the results of a case study was then performed through a data-driven and criteria-based evaluation (Phase 5). The case study addressed a particular type of team composition problem in an academic unit of a higher education institution. Finally, the documentation for each class and the user manual and technical aspects of the ontology was generated (Phase 6). The next section now presents the details of these six phases.

## 3. Results

In summary, the methodology we follow for development includes ontology definition, conceptualization, formalization, implementation, evaluation, and documentation. Here we describe each in turn, giving specific technical elaborations.

### 3.1. Definition

The objective of a requirements specification is to produce a formal or informal description of the rationale behind the development of an ontology and to elucidate its potential uses (Fernández-López et al. 1997). This can be done by documenting this information in natural language, by using specification questions, and by developing intermediate representations. This study used the following set of specification questions adapted from Fernández-López et al. (1997) to help determine the ontological rationale. This is augmented by a use case diagram in response to the “intended use” question.

- What is the purpose? The objective of the ontology is to facilitate the integration of metareasoning processes into advanced intelligent systems.
- What is the scope? The ontology will include information on the processes related to metareasoning, such as allocation deliberation time, allocation evaluation effort, detection of reasoning failures, meta-explanations, introspective monitoring and metalevel control.
- Who are the intended end users? Users include research groups in cognitive science, artificial intelligence, and cognitive computing. Although the proposed ontology addresses general topics of meta-reasoning, our focus is the application in educational settings, mainly in solving academic problems in higher education institutions.
- What is the intended use? The main functional roles include the ability to model meta-reasoning problems that can occur in intelligent systems in terms of monitoring and controlling cognitive processes. The ontology is useful for reducing the discrepancies between the data structures required in different components of the meta-reasoning process. Discrepancies between data structures and language syntax make it even more difficult to exchange information between meta-reasoning models, leading to considerable information deviations when data flows are connected through the different designed components. Knowledge sharing among researchers, academics, and developers can be facilitated by having an ontology for the meta-reasoning domain. This is because the ontology reduces the ambiguity of terms and has a controlled vocabulary. The ontology provides a semantic basis for communication between designers, academics, and researchers so that designers share a common understanding of knowledge.

Existing ontologies and publications in specialized databases were the main resources identified as sources of information for the extraction of terms related to meta-reasoning. Science Direct, IEEE Xplorer, ACM, Springer and IGI Global were searched using the keywords “metareasoning”, “meta-reasoning”, ”metareasoning problem”, ”meta-level control”, ”metacognition”, “anytime algorithm” and combinations of these keywords. Similarly, a complementary search was conducted in Google Scholar and Scopus.

### 3.2. Conceptualization

Following the definition of the ontology requirements specification, the next step is to describe how the domain knowledge was acquired and formalized. The main steps in this phase include:

(i) Listing the relevant terms in the ontology;
(ii) Defining classes;
(iii) Defining class properties with specifications for their range and domain (Noy and McGuinness 2001).

#### 3.2.1. Listing the Relevant Terms in the Ontology

Knowledge relevant to the ontology was initially acquired through a detailed review of existing studies on metareasoning ontologies. Two key publications proved especially valuable. Table 1 lists the main terms that appear in existing ontologies from this literature in the metareasoning domain.

Given these terms as a base, we extended them by reviewing a select set of key papers about metareasoning and related topics. Table 2 shows the literature reviewed, including computational metacognition, introspective monitoring, and anytime planning algorithms.

**Figure 2:**
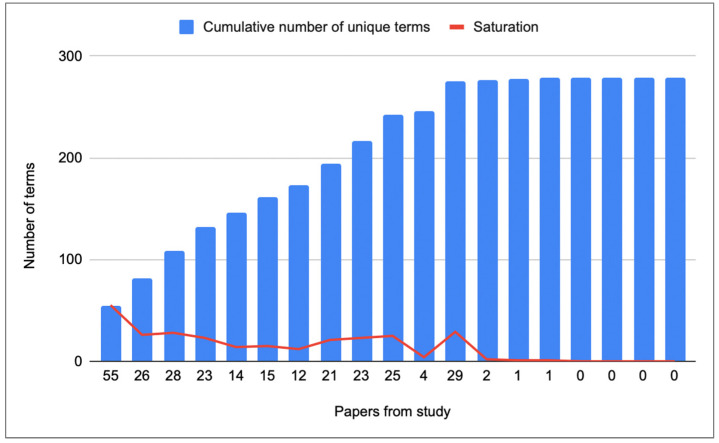
Saturation level as the number of novel terms added by a subsequent article. The papers were processed in the same order as in Table 2. The x-axis corresponds to the new terms provided by each paper after analyzing them in order.

The concept of data saturation was taken as an indicator of when to stop the literature review process. Data saturation occurs when new information is not obtained with additional data collection effort (Fusch and Ness 2015). Figure 2 presents how data saturation was achieved in this study. In this figure, a decreasing return trend (e.g., redundant data) exists in the number of unique information attributes (used to construct concepts) identified from the reviewed papers. After article number 15, no additional unique information elements were identified.

#### 3.2.2. Defining Classes

The classes of the ontology are drawn from the term list presented in Section 3.2.1. We selected terms that describe objects that have an independent existence or represent a collection of individuals or objects. The selected terms define the ontological classes and constitute the nodes in the class hierarchy (see Table 3).

#### 3.2.3. Define Class Properties with Specification of the Range and Domain

This section describes the relationships and properties of some class examples, but due to the large number that has been identified, we do not report all in the ontology. In cognitive systems with a metalevel-based architecture, the function of the object level (i.e., the reasoning level) is to recognize and solve problems or situations in the environment where the system operates. In contrast, the main function of the meta-level is to monitor and control the reasoning and learning processes that take place at the object level (Cox and Raja 2011; Nelson 1990). The objective of monitoring and control is to achieve more effective results in the processes carried out at the object level.

A meta level has a problem space and a metareasoner (see Table 4). The problem space represents a collection of metareasoning problems and all possible paths to solving them. The metareasoner is the computational algorithm that executes all metareasoning tasks.

Meta-reasoning tasks are a particular type of metacognitive task whose purpose is to monitor and control the reasoning processes that take place at the object level (again, see Table 4). Some properties of meta-reasoning tasks are the input parameters, the output, the necessary conditions for its execution, the effects of its execution, as well as the event and completeness states. Table 4 shows the properties and domain range of two classes in the ontology.

### 3.3. Formalization

**Figure 3:**
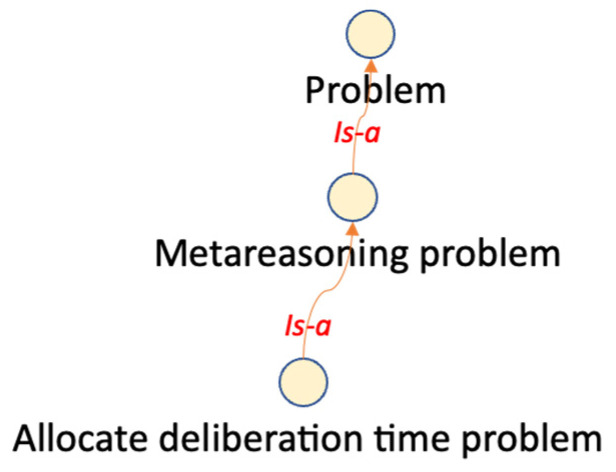
Class hierarchy according to (Conitzer and Sandholm 2003). Figure elaborated by the authors.

**Figure 4:**
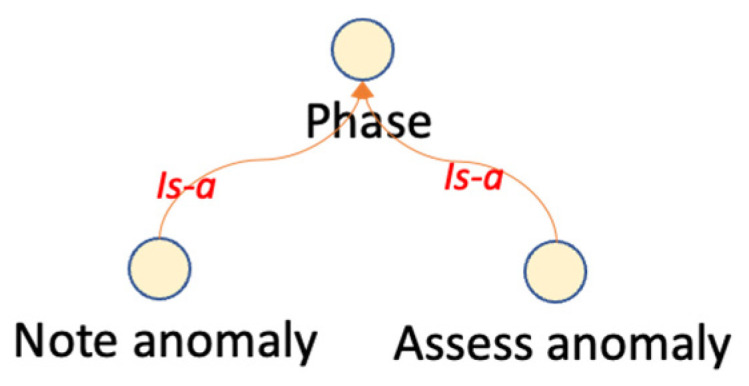
Class hierarchy extracted from a previous ontology in (Schmill et al. 2007). Figure elaborated by the authors.

Hierarchical classifications use predefined semantic taxonomies (Silla and Freitas 2011). A taxonomy contains only one root class and defines the “Is-A” relationships between classes. An “Is-A” relationship is transitive and asymmetric. Some ontological hierarchies were generated from the explicit descriptions in the reviewed papers, as can be seen in Figure 3 and Figure 4.

Other class hierarchies were drawn from existing ontologies, for example:

Finally, other ontological hierarchies arose from the process of concept abstraction. For example, reasoning tasks and metareasoning tasks abstract the task class.

In this ontology, the information about the metareasoning process is modeled according to the standard vocabulary that facilitates a better semantic interpretation that can resolve ambiguities in the terms used in the different data sets, metareasoning applications, or when a little additional knowledge can lead to the discovery of new relationships between terms. In this sense, this ontology is designed to semantically represent a rich and complex knowledge about the domain of metareasoning in intelligent systems.

The semantic relationships in the ontology are given through hierarchical relationships of type Is-Un. When a relationship of this type is established between two classes, one is a superclass, and the other is a subclass. The subclass shares the structure and behavior of the superclass. In the same way, the “HAS” relationships have been implemented, which are structural and describe a set of links, which describe composition connections between the terms.

The ontology has mechanisms to verify the consistency of this knowledge and make explicit the implicit knowledge that can be generated in the monitoring and control processes of reasoning in intelligent systems.

### 3.4. Implementation: The Integrated Metareasoning Ontology (IM-Onto)

This section presents the technical details of the Integrated Metareasoning Ontology (IM-Onto). To make an ontology machine-readable, it is important to represent it in a formal computational language (Noy and McGuinness 2001). Hence, IM-Onto was implemented using OWL/RDF (Allemang et al. 2005) in the Protégé environment (Horridge et al. 2019).

The advantage of using OWL/RDF is that it allows for richer semantic expressions of concepts, their attributes, and the relationships between them. At the same time, the use of the OWL/RDF model supports improved interoperability or connection of information silos with the added merit of being able to generate implicit semantic inferences based on defined relationships (Horridge et al. 2019). In the context of this study, the use of this model for linking information enables a machine-readable representation (through uniform resource identifiers) that also solves a vocabulary problem in this domain. In the context of this study, the use of this model for metareasoning enables a machine-readable representation (through uniform resource identifiers) that also solves a vocabulary problem in this domain.

**Figure 5:**
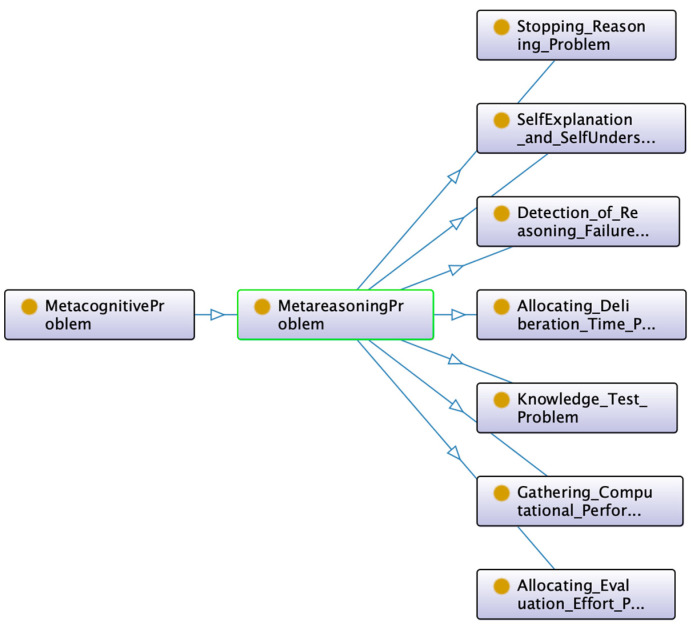
Subontology hierarchy.

IM-Onto consists of seven sub-ontologies that map to key metareasoning problems. These seven are as follows: the Allocating Deliberation Time Problem (ADTP); the Allocating Evaluation Effort Problem (AEEP); the Knowledge Test Problem (KTP); the Stopping Reasoning Problem (SRP); the Gathering Computational Performance Data Problem (GCPDP); the Detection of Reasoning Failure Problem (DRFP); and finally, the Self-Explanation and Self-Understanding Problem (SE&SUP). Figure 5 shows the hierarchy of these subontologies in IM-Onto.

To facilitate the ontology reading process and to differentiate an ontology concept or a concept-relation property from a regular term, the model uses the following.

- CAPITAL LETTERS denote concepts defined in the ontology. For example, ALLOCATE_DELIBERATION_TIME and OBJECT_LEVEL are important concepts and are further described in the ontology term detailed description.
- *Italics letters* refer to a property of a relation between two or more ontology concepts. For example, *has_problem_solution* is a property that relates the ontology term PROBLEM and SOLUTION.

#### 3.4.1. The Allocating Deliberation Time Problem (ADTP) Subontology

**Figure 6:**
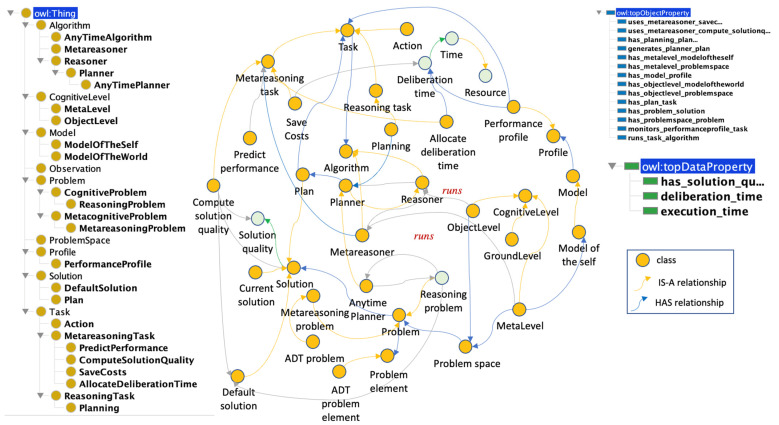
ADTP subontology.

In this section, the setting where an agent must allocate its deliberation time across different problems is represented. Figure 6 portrays the entire sub-ontology, including the ALLOCATE_DELIBERATION_TIME class with its relations. However, the concept of a METAREASONING_PROBLEM is presented first due to its key role in the overall metareasoning process.

A METAREASONING_PROBLEM is a class that represents the problem an agent has of monitoring and controlling the progress of its own reasoning and problem-solving activities and regulating the time and effort spent on them (Ackerman and Thompson 2017). This definition is a good example of the creation of inferred abstract concepts such as the concept PROBLEM. The PROBLEM class is considered part of the core of the ontology because it is used by all the sub-ontologies. In the proposed design, every PROBLEM is part of a PROBLEM_SPACE and has a SOLUTION. The meta-level analyzes the DEFAULT_SOLUTION for potential anomalies or optimizations after the object level generates it.

The ADT_PROBLEM class is a particular type of problem for which the main elements are part of the ADT_PROBLEM_ELEMENT class and are listed below: PERFORMANCE_PROFILE, ANYTIME_ALGORITHM and METAREASONING_TASK.

- A PERFORMANCE_PROFILE generally represents a vector with the quality of the solutions of an algorithm that is monitored in reasoning time intervals.
- ANYTIME_ALGORITHM is a class that represents an anytime algorithm whose quality of results gradually improves as computation time increases and can return a valid solution to a problem even if it is interrupted before completion. This kind of algorithm offers a tradeoff between solution quality and computation time, which is expected to find better solutions the longer it keeps running. In the context of this work, an anytime algorithm works to solve a REASONING_PROBLEM.
- The METAREASONING_TASK class refers to tasks that are carried out in the META_LEVEL, and its objective is to monitor and control the reasoning processes that are carried out at the OBJECT_LEVEL. The main metareasoning tasks in this problem are COMPUTE_SOLUTION_QUALITY, PREDICT_PERFORMANCE, ALLOCATE_DELIBERATION_TIME and SAVE_COST.

In this type of problem, a system has several algorithms that can be executed in parallel. Each algorithm solves one instance of a problem, but in some circumstances where execution time is limited, the metalevel must select a subset of algorithms to execute.

For example, a shipping company may have three algorithms to calculate three routes to three different cities. Each route is optimized by an anytime algorithm, but if a time restriction is included, for example, the algorithm only has a third of the normal execution time to optimize the route and save costs, the metalevel reviews the performance profile of the three algorithms in the required time (1/3) and selects the algorithm with the best-expected performance in the required time.

#### 3.4.2. Allocating Evaluation Effort Problem Subontology

In this section, we represent the setting where an agent is faced with multiple options (actions) from which it eventually must choose one. The agent can use deliberation or information gathering to evaluate each action, but with a given limited time, it must decide which ones to evaluate.

**Figure 7:**
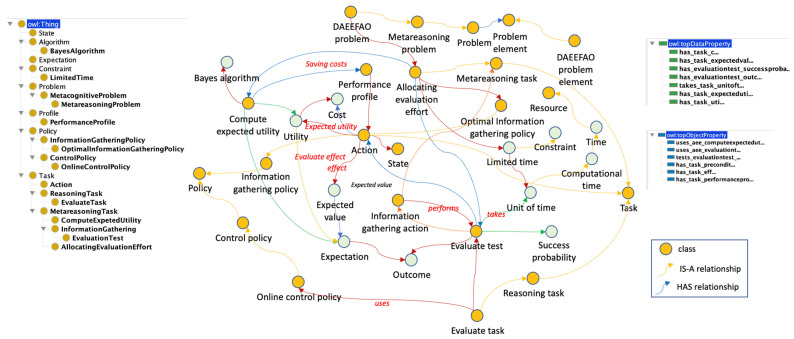
The AEEP subontology.

Figure 7 shows the entire sub-ontology, including the Dynamically Allocating Evaluation Effort Across Option class (EVALUATION_EFFORT_PROBLEM) with its relations.

The EVALUATION_EFFORT _PROBLEM class is a particular type of problem whose main elements are part of the DAEEAO_PROBLEM_ELEMENT class and are listed below: ACTION, EVALUATION_TEST, PERFORMANCE_PROFILE and METAREASONING_TASK.

- Class ACTION represents a set of actions that an agent could evaluate.
- EVALUATION_TEST allows to verify the history of events or failures of the execution of an action. This can bring savings to the ACTION selection evaluation effort.
- The main metareasoning tasks in this problem are ALLOCATING_EVALUATION_EFFORT, COMPUTE_EXPECTED_UTILITY, and EVALUATE_TEST.

In this problem, the metareasoning process has already selected an algorithm to solve a problem, but this algorithm has several different actions to perform the same task. The meta-level must now recommend an action for the object level to perform a specific task, and the recommendation is made based on the highest EXPECTED_UTILITY. In this sense, given the current conditions of the system, the meta-level recommends that the algorithm execute the action with the best-expected utility. The EXPECTED_UTILITY is predicted based on the performance profile of an action, which is stored in the MODEL_OF_THE_SELF.

For example, an application that forms work teams may have three ways of selecting candidates to be part of the teams: (i) randomly, (ii) a person with experience in the position, and (iii) maximum score in tests of attitude. The metareasoning problem arises when the restriction is included, and there is only time to evaluate some of the available actions. Then the META_LEVEL must allocate the evaluation effort only to the actions that have the best EXPECTED_UTILITY. In this sense, the META_LEVEL monitors the PERFORMANCE_PROFILE of previous evaluations of each action in previous similar cases. The ACTION with the best EXPECTED_UTILITY will be chosen for evaluation, and the PERFORMANCE_PROFILE of the action will be modified to reflect the QUALITY of its output.

#### 3.4.3. The Knowledge Test Problem (KTP) Subontology

In this section, we represent the problem whereby an agent has only one item to evaluate, but it must choose the order of deliberation or information-gathering actions for doing so. However, the system is not able to identify the status of the item to be evaluated and needs to run a series of tests (knowledge tests) to disambiguate the status of the item, but the system does not have enough time to run all the tests.

**Figure 8:**
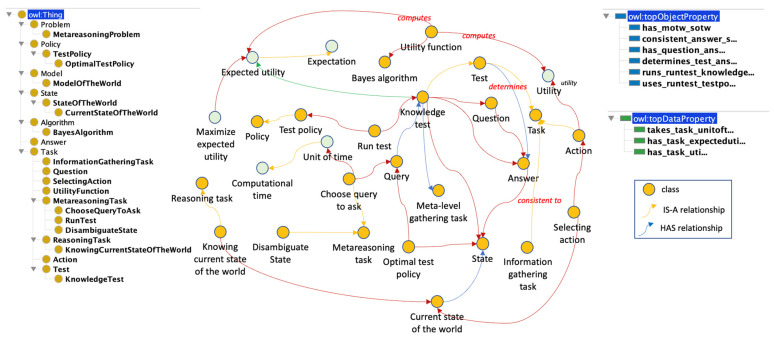
The KTP subontology.

Figure 8 shows the entire sub-ontology, including the class STATE_DISAMBIGUATION_PROBLEM with its relations.

The STATE_DISAMBIGUATION_PROBLEM class is a particular type of METAREASONING_PROBLEM, whose main elements are part of the STATE_DISAMBIGUATION _PROBLEM_ELEMENT class and are listed below: KNOWLEDGE_TEST, QUERY, ANSWER and METAREASONING_TASK.

- The KNOWLEDGE_TEST class determines the answers to a set of questions to determine the current state of the world.
- Class QUERY represents a set of questions to determine the current state of the world.
- Class ANSWER contains the output of the queries.

In this problem, three sub-classes exist for the METAREASONING_TASK class. They are CHOOSE_QUERY_TO_ASK, DISAMBIGUATE_STATE, and METALEVEL_GATHERING_TASK.

This type of METAREASONING_PROBLEM occurs when the OBJECT_LEVEL is not able to disambiguate the state of the world given the current observations. For example, a ship traveling at high-speed stops abruptly because it cannot identify what type of obstacle it has in front of it, whether it is a cloud, another ship, or a building. To identify the type of obstacle, the OBJECT_LEVEL must perform a series of KNOWLEDGE_TESTs, and, depending on the result of the test, it must act. If the obstacle is a cloud, then the ACTION to follow is to move forward and go through the cloud. If the obstacle is another ship, the ACTION is to decelerate and change altitude until the ship has passed, and if the obstacle is a building, the ACTION is to change course. However, if the time to perform the KNOWLEDGE_TESTs is limited and not all can be performed, then the META_LEVEL must select which KNOWLEDGE_TEST to do to change the knowledge about the state of the world. A knowledge test is based on a series of questions and answers that clarify the state of the world. The questions are predefined by the designers, but in complex systems, they can be self-generated from the agent’s observations, expectations, and prior knowledge. This case of metareasoning is very particular because it requires that the systems or agents act based on their knowledge and not on the information of their internal state; most systems are not designed for this purpose.

#### 3.4.4. The Stopping Reasoning Problem (SRP) Subontology

**Figure 9:**
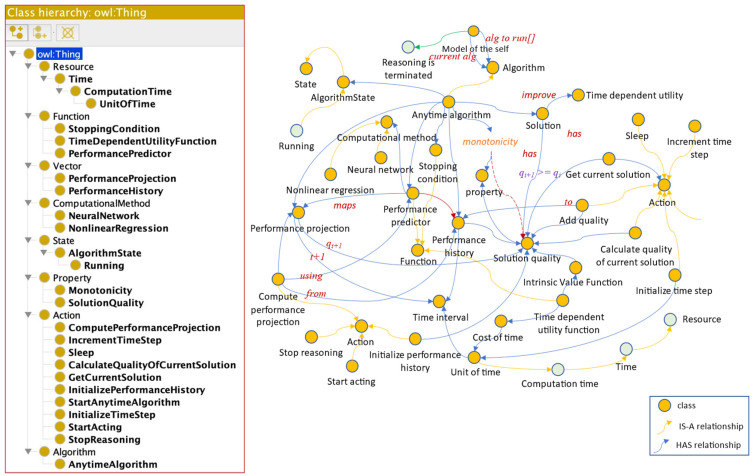
The SRP subontology.

Stopping reasoning is the most basic decision of the metareasoning process in intelligent systems. Figure 9 shows the entire sub-ontology, including the STOPPING_REASONING_PROBLEM class with its relations. The STOPPING_REASONING_PROBLEM class is a particular type of METAREASONING_PROBLEM, whose main elements are part of the STOPPING_REASONING_PROBLEM_ELEMENT class and are listed below: TIME_DEPENDENT_UTILITY, SOLUTION_QUALITY, PERFORMANCE_PREDICTOR, PERFORMANCE_PROFILE_HISTORY, PERFORMANCE_PROFILE_PROJECTION and METAREASONING_TASK.

- A TIME_DEPENDENT_UTILITY represents the utility of a solution computed by an anytime algorithm.
- SOLUTION_QUALITY represents the quality of the solution to a problem. In this case, a solution is generated by an algorithm to solve the current problem.
- A PERFORMANCE_PROFILE_HISTORY represents the past performance of an anytime algorithm as a vector of solution qualities.
- A PERFORMANCE_PROFILE_PROJECTION represents the future performance of an anytime algorithm as a vector of solution qualities.
- PERFORMANCE_PREDICTOR is a function that maps a PERFORMANCE_PROFILE_HISTORY to a PERFORMANCE_PROFILE_PROJECTION.

In this problem, the sub-classes of METAREASONING_TASK are PREDICT_PERFORMANCE, INTRINSIC_VALUE_FUNCTION, TIME_DEPENDENT_UTILITY_FUNCTION, COMPUTE_PERFORMANCE_PROFILE, and INIT_PERFORMANCE_PROFILE_HISTORY.

The interruption of the reasoning process is considered the most basic decision of the metareasoning process. In anytime systems that need to make decisions with limited resources, the metareasoning process is responsible for making the decision to continue reasoning or act on the current plan. For this, various techniques have been proposed in the literature, but the most common is to make the decision based on a TIME_DEPENDENT_UTILITY_FUNCTION. This is because the passage of time has a cost in itself, so a SOLUTION obtained in a reasonable time is what is desired. For example, going back to the case of the formation of teams, it is better to form a team with an EXPECTED_UTILITY of 0.7 using 5 min of reasoning than to form a team with an EXPECTED_UTILITY of 0.8 but the reasoning for nine hours (the EXPECTED_UTILITY is given by the average of the evaluation of all the skills of the team members).

The algorithm evaluates whether it is worthwhile to perform another reasoning cycle or if, on the other hand, the cycle should be stopped in any iteration, and then the CURRENT_PLAN is executed. A technique for the evaluation is to implement a predictor (PERFORMANCE_PREDICTOR) or function that predicts the EXPECTED_UTILITY of the solution in the next reasoning cycle; if the EXPECTED_UTILITY is positive, the algorithm continues, but if the utility is negative, then the META_LEVEL stops the process of reasoning.

#### 3.4.5. The Gathering Computational Performance Data Problem (GCPDP) Subontology

**Figure 10:**
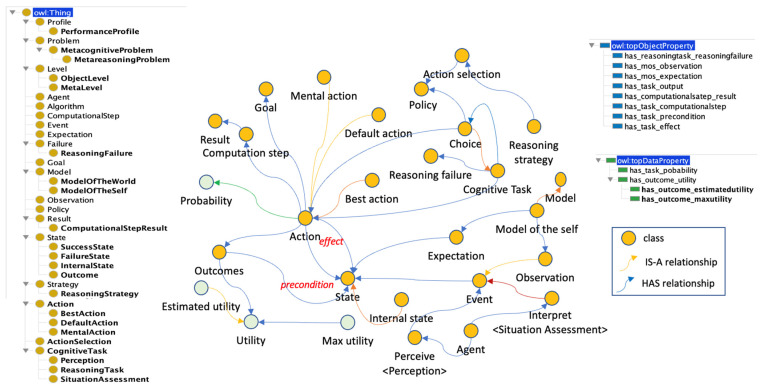
The GCPDP subontology.

Information gathering is a fundamental task for making metacognitive control decisions. Information is collected to store information in models. If the model is about the state of the world, then it is stored in the object level, and if the model is of the internal processes, then it is stored in the meta-level. Figure 10 shows the entire sub-ontology, including the INFORMATION_GATHERING_PROBLEM class with its relations.

The INFORMATION_GATHERING_PROBLEM class is a particular type of METAREASONING_PROBLEM, the main elements of which are part of the INFORMATION_GATHERING_PROBLEM_ELEMENT class and are listed below:

- MODEL_OF_THE_WORLD is an internal model that stores information related to the perception history that describes the state of the environment or world that is perceived by the system.
- MODEL_OF_THE_SELF is a dynamic model of the object level. This model is part of the meta-level and contains updated information on the current state of the reasoning processes that are carried out at the object level.

The following are the sub-classes of COGNITIVE_TASK in this problem: PERCEPTION, REASONING_TASK, and SITUATION_ASSESSMENT.

Monitoring of reasoning processes has been less studied than meta-level control. In this sense, the META_LEVEL executes a series of tasks to gather information on the state of the reasoning processes that are carried out at the OBJECT_LEVEL. The information collected is stored in performance profiles that make up the MODEL_OF_THE_SELF. The model of the self allows the META_LEVEL to have an updated view in real time of the internal states of the reasoning processes. This information is used for learning processes and for decision-making in the meta-level control.

#### 3.4.6. Detection of Reasoning Failure Problem (DRFP) Subontology

**Figure 11:**
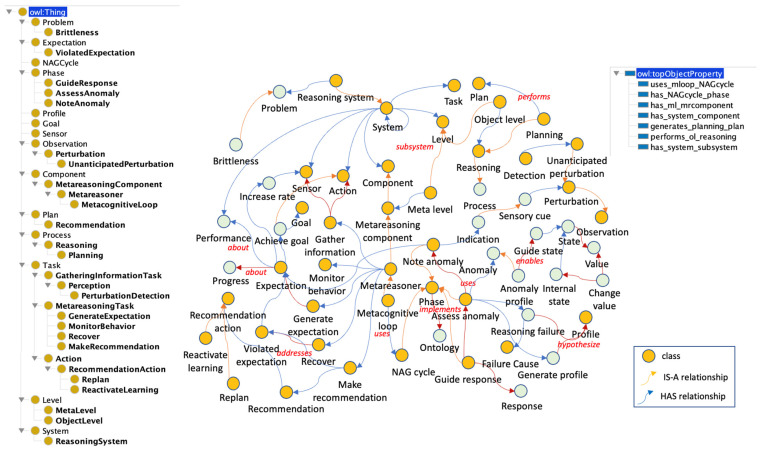
The DRFP subontology.

Stopping reasoning is the most basic decision of the metareasoning process in intelligent systems. Reasoning about reasoning failures is an important research topic in metareasoning. In this sense, at some level of abstraction, the ways in which a system can fail are finite. Thus, the knowledge of how systems fail and how to recover from these failures must be represented. Figure 11 shows the full sub-ontology, including the class SOLVING_REASONING_FAILURE_PROBLEM along with its relations.

The SOLVING_REASONING_FAILURE_PROBLEM class is a particular type of METAREASONING_PROBLEM whose main elements are part of the SOLVING_REASONING_FAILURE _PROBLEM_ELEMENT class. These elements are listed below:

REASONING_FAILURE, ANOMALY, EXPECTATION, RECOMMENDATION, and GOAL.

Furthermore, in this problem, the METAREASONING_TASK class has three sub-classes. They are MAKE_RECOMMENDATION, ANOMALY_DETECTION, and GENERATE_EXPECTATION.

During the reasoning process, errors such as incomplete tasks or unexpected results can occur that can affect the execution of critical tasks for an intelligent system. These errors are called reasoning failures and are represented in the class REASONING_FAILURE. An unexpected result is considered an ANOMALY in the performance of the task or process. Many designers of intelligent systems establish metareasoning points because monitoring and controlling all the reasoning processes of the system can be costly in terms of resource consumption. A METAREASONING_POINT is a TASK or process that is considered important for the operation of the system, and therefore, it is necessary to monitor its performance. The META_LEVEL keeps a record of the decisions made at the metareasoning points to analyze these traces of reasoning in the event of failures. When a REASONING_FAILURE is detected, then the META_LEVEL identifies the failure and proceeds to fix it based on the knowledge acquired from previous failures. The detection of a REASONING_FAILURE is carried out by monitoring the states of the metareasoning points. The most common failures are excessive processing time, excessive pause time waiting for a response from another task, and unexpected results that produce violations of expectations (e.g., VIOLATED_EXPECTATION). Performance expectations are generated from historical data and are contrasted against current observations of the performance of the task or process. After a REASONING_FAILURE is detected, the META_LEVEL generates a GOAL to solve the failure at the OBJECT_LEVEL. The GOAL is embodied in a RECOMMENDATION that can include any of the following options: abort the task, pause the task, reconfigure the task or resume the task. Finally, the SOLUTION is stored for future reference.

#### 3.4.7. Self-Explanation and Self-Understanding Problem (SE&SUP) Subontology

**Figure 12:**
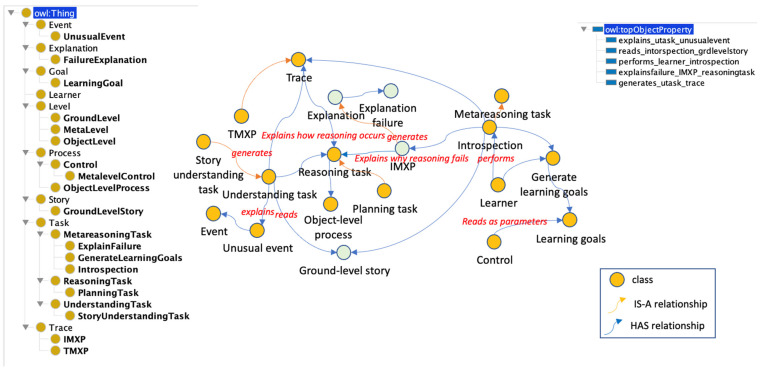
The SE&SUP subontology.

If the reasoning that is performed at the object level (and not just its results) is represented in a declarative knowledge structure that captures the mental states and decision-making sequence, then these knowledge structures can themselves be passed to the metalevel for monitoring. Figure 12 shows the entire sub-ontology, including the SELF_METAEXPLANATION_PROBLEM class with its relations.

The SELF_METAEXPLANATION_PROBLEM class is a particular type of METAREASONING_PROBLEM, whose main elements are part of the SELF_METAEXPLANATION_PROBLEM_ELEMENT class and are listed below: REASONING_TRACE, IMXP, TMXP, FAILURE_EXPLANATION, and LEARNING_GOAL. In this problem, the sub-classes of METAREASONING_TASK are STORY_UNDERSTANDING_TASK, EXPLAIN_FAILURE, GENERATE_LEARNING_GOAL, and INTROSPECTION.

The SELF_METAEXPLANATION_PROBLEM class represents the METAREASONING_PROBLEM that occurs when the OBJECT_LEVEL is unable to generate an EXPLANATION for an ANOMALY in the performance of the reasoning process when a STORY_UNDERSTANDING_TASK is running. With STORY_UNDERSTANDING_TASK a system must be able to reason introspectively about how to complete a task and what specific pieces of knowledge it needs to improve its performance at that exact moment to effectively learn the current task that is running at the object level. The META_LEVEL reads the REASONING_TRACE that contains the decisions made and the internal state of the system at the time of the decision. The META_LEVEL then runs a meta-understanding process (e.g., EXPLAIN_FAILURE) based on a REASONING_TRACE to understand the cause of the REASONING_FAILURE and why the OBJECT_LEVEL could not explain it. Once the cause is understood, the META_LEVEL generates a LEARNING_GOAL to recommend the actions that the OBJECT_LEVEL should take. The reasoning trace, the generated goals, the FAILURE_EXPLANATION and the recommendations are stored for future reference.

#### 3.4.8. Improving the Ability of the Approach to Generalize to New (or Existing but Unstudied) Problems

The proposed ontology can be extended beyond the seven subontologies to capture new meta-reasoning problems that were not covered by this research. In this sense, it may be the case that different meta-reasoning problems induce new subontologies. To prevent new subontologies from generating counterproductive effects in terms of sharing knowledge in an easier way, a version-based approach will be adopted. In this way, when exchanging knowledge between applications or research teams, the use of different versions of the ontology can be avoided.

**Figure 13:**
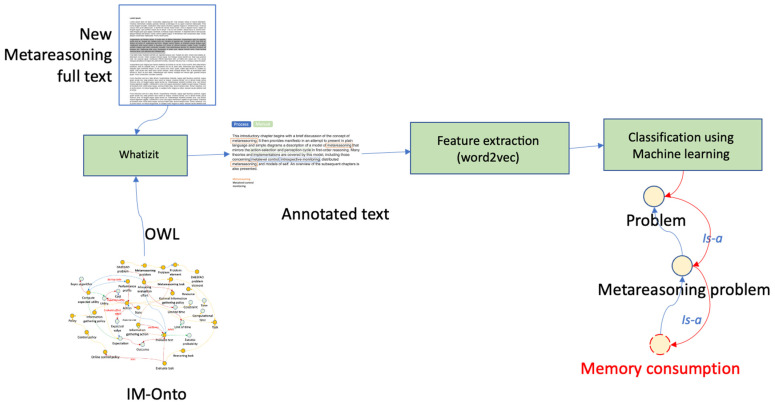
Semi-automated approach to creating new subontologies based on (Althubaiti et al. 2020).

In relation to the creation of new subontologies and their relationship with existing ones, two different approaches can be taken to preserve the integrity of the ontology. Researchers can opt for a manual approach following the method described in this paper; the resulting subontology will be evaluated by experts to be integrated into IM-Onto. The second is a semi-automated approach based on (Althubaiti et al. 2020), in which the term extraction process for the new subontologies is carried out through an analysis of the corpus contained in documents that describe meta-reasoning problems. This approach uses a semi-automated approach to recognize the mention of IM-Onto classes in the text. The approach is based solely on labels and synonyms of the classes within the IM-Onto ontology and can be used to determine whether a word refers to an IM-Onto class; see Figure 13.

First, the IM-Onto ontology is obtained in Web Ontology Language (OWL) format, and the list of class labels and synonyms is extracted from the ontology, also using a body of text (article or description of the new meta-reasoning problem) as input to the process. Text mining tasks are performed with the Whatizit tool (Rebholz-Schuhmann et al. 2008). Word embeddings (i.e., vector space encodings of the contexts in which a word occurs) are then generated for all words in the text corpus, and a supervised machine learning model is trained to classify whether a word refers to a class in IM-Onto or not (using the ontology words and their synonyms as positive training examples and all others as negative examples). This approach broadly identifies terms that refer to classes within the domain of a meta-reasoning problem (according to the number of classes there are in the ontology). The method generates “seed” words in the text and then uses these seeds first to generate context-based features (via Word2Vec (Mikolov et al. 2013), and it uses these context-based features in a supervised machine learning classifier.

### 3.5. Evaluation and Case Study

This section evaluates the use of IM-Onto in an application to solve a real-world problem. The resulting case study enables the use of automatic consistency checking and multiple performance evaluations (i.e., task-based, data-driven, and criteria-based evaluation). In this sense, the ontology evaluation consists of automated consistency checking (ontology verification) and task-based, data-driven, and criteria-based evaluations.

#### Running Example: The Team Allocation for Internship Programs (TAIP)

**Figure 14:**
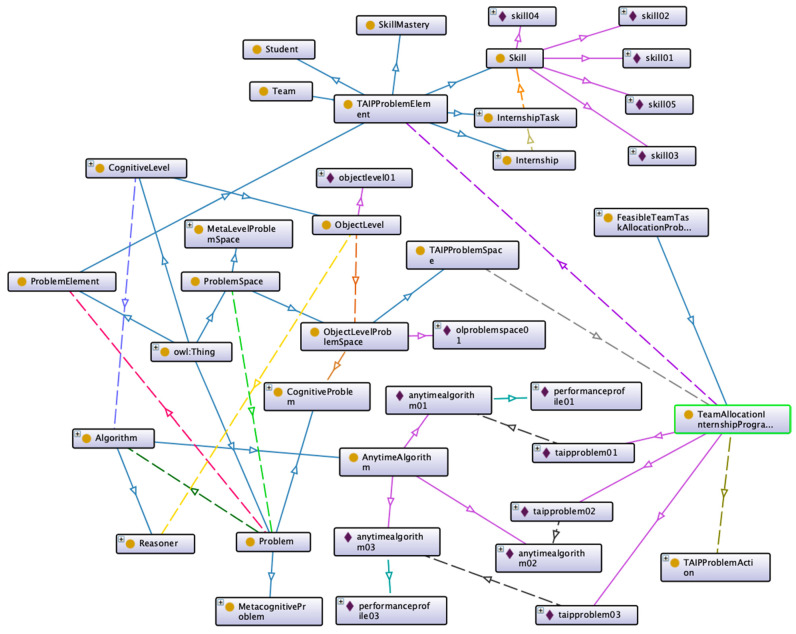
Representation of knowledge generated in the process of monitoring and control of the Team Allocation for Internship Programs Problem (TAIP).

IM-Onto was applied to a real-world problem to demonstrate its practical use in supporting metareasoning problems. This case study has been based on the assignment of internships as a degree option in the undergraduate program of Computer Science (Licenciatura en Informática) at the University of Córdoba—Colombia. Allocating student teams to internship programs is a particular type of team composition problem (Andrejczuk et al. 2019). The problem has been selected because different studies (Georgara et al. 2020a) have used anytime algorithms to find approximate solutions to team allocation in the education context based on the Feasible Team-For-Task Allocation Problem (FTAP). FTAP considers the problem of putting together teams of students suitable for internship tasks in companies and institutions, given that it is increasingly common for students to spend some time doing internships in a company as part of their study plan. Figure 14 shows the representation of knowledge generated in the process of monitoring and control of the Team Allocation for Internship Programs Problem (TAIP).

The undergraduate program of Computer Science has several internship programs to which students can apply. An internship program is characterized by the skill requirements for students and the limitations of team size. In the internship program selected for the case study, a student is characterized by their skills and their level of mastery of each skill. Students must meet 5 skill requirements. These are (a) principles of image theory and photography, (b) STEAM (science, technology, engineering, arts, and math) skills, (c) web development, (d) fluency in the Spanish language, and (e) protégé video production, whereas the required team size is 3 members. Each student has a required mastery level in each of the skills.

Figure 14 shows a partial view of the ontology that represents the knowledge generated in the process of monitoring and control of the Team Allocation for Internship Programs Problem (TAIP) (Georgara et al. 2020b), and a knowledge base is constructed in this case study by adding instances to the classes defined in the ontology.

The steps followed for the implementation of an intelligent agent based on the ontology specifications are presented below.

**Figure 15:**
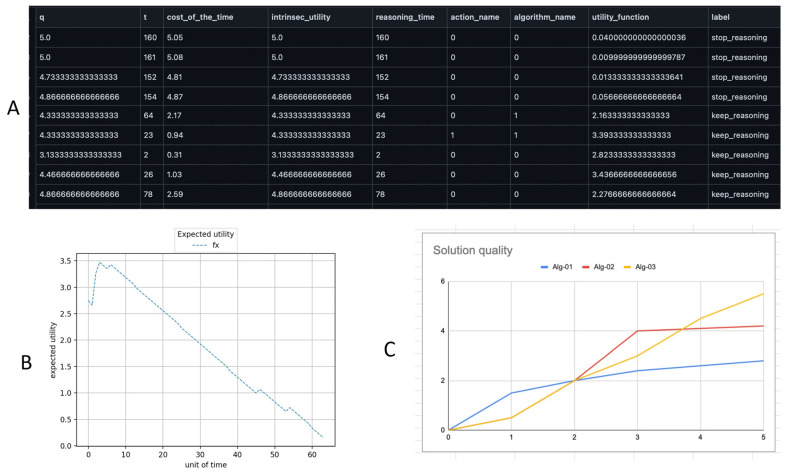
Data resulting from the validation process of TAIP. Section (**A**): dataset generated in a stopping reasoning problem. Section (**B**): behavior of the time-dependent utility function used to predict the stopping of the reasoning process. Section (**C**): performance profiles of three algorithms that solve TAIP, which ADTP uses to select a subset of algorithms according to the problem constraints. The x-axis represents reasoning loops.

1. A Python package was developed that contains the classes common to the 7 types of problems addressed in this article. In this package, a basic cognitive system is made up of two cognitive levels, as specified in the ontology. A Beta version of the package is available at: https://github.com/dairdr/carina (login is required).
2. A polymorphic meta-reasoner was created at the meta-level, which monitors the object level by accessing the data that is updated in the model of the self (MoS); this model is designed according to the MODELOFTHESELF class. The monitoring and gathering of information are done through the MoS, which is updated in real-time from the object level. The MoS stores the profiles of the cognitive tasks that are executed at the object level; this is done automatically and does not require human intervention. Figure 15 shows a dataset based on the performance profile of the object-level reasoner and the history of a stopping reasoning problem (SRP). The meta-level uses the dataset to train SRP using a random forest algorithm; see Figure 15, section A. The MoS is stored in the working memory of the cognitive system, serving as a bridge between the object level and the meta-level. The meta-reasoner runs in parallel with the object level and analyzes the reasoning traces of the cognitive task profile. The meta-reasoner analyzes the data using a random forest algorithm to select the method to execute according to the meta-reasoning problem detected. Profiles store data about cognitive tasks such as start time, execution time, output quality, and data that are common to any task executed at the object level. In this sense, the scaling of the meta-reasoner is facilitated, encompassing new models due to its polymorphic design.
3. A cognitive system with two cognitive levels was designed: the object level and the meta-level. The object level was configured according to the IM-Onto object level class specifications. Where the problem or cognitive task performed by the object level was defined considering the REASONINGPROBLEM class, then the elements of the problem were added according to the PROBLEMELEMENT class.
  In this case study, the object level is based on three anytime algorithms that are monitored and controlled by a meta-level until a suitable solution is found in cost and time. An example of the implementation of the FTAP problem is available at: https://github.com/dairdr/carina/blob/master/miscellaneous.py (login is required).
4. The system was configured with three algorithms to induce some meta-reasoning problems to observe the behavior of the meta-level. In this sense, for TAIP, one algorithm randomly selects the members of the team, another selects the most qualified members for each competition and thus assembles the team, while another algorithm receives parameters that restrict the selection; for example, if the average skill proficiency of a selected team has a student with low performance, then the algorithm replaces the student. The meta-level selects the best algorithm profile according to a set of constraints stipulated in the problem configuration, such as deliberation time and algorithm performance. Figure 15 shows some outcomes resulting from the validations of the system. Section A shows a subset of features obtained from the profiles of the cognitive tasks that are stored in the MoS and are used by the meta-level for monitoring and control; in this case, it is a training dataset for the problem of stopping the reasoning process. Section B shows the behavior of the time-dependent utility function of the algorithm that is running at the object level, which is used to predict the stopping of the reasoning process. Section C presents the profiles of the three algorithms with respect to the behavior of the time-dependent utility function.

### 3.6. Ontology Evaluation

This section describes four approaches for the evaluation of IM-Onto. First, the automated consistency check approach is used to evaluate the internal consistency of the ontology. Second, the task-based assessment evaluates that IM-Onto can accomplish the competency tasks defined in the specification by using completeness questions. Third, data-driven evaluation rigorously tests the integrity and conciseness of the ontology. Finally, criteria-based assessment further addresses the ontology clarity metric.

#### 3.6.1. Automated Consistency Checking

**Figure 16:**
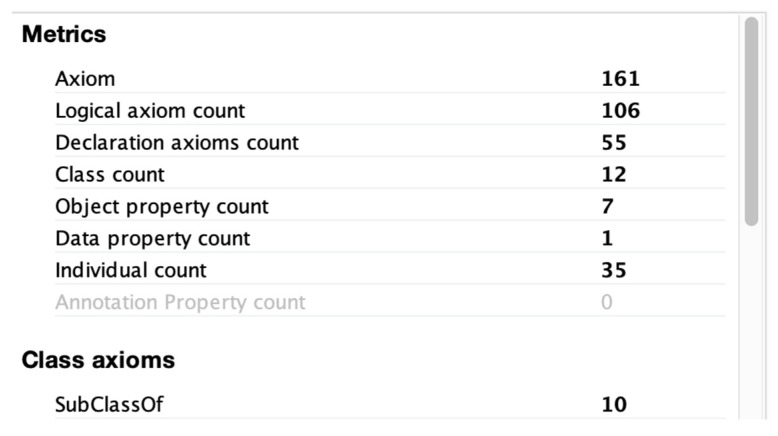
Results of verification cycle.

The consistency check of an ontology is used to confirm that there are no contradictory facts according to descriptive logic (DL). IM-Onto was evaluated using the Pellet reasoner. Pellet is an OWL-DL reasoner built into the open source Protégé with reasoning support for individuals (instances), cardinality constraints, user-defined data types, sub-property axioms, reflexivity constraints, symmetric properties, and disjoint properties (Sirin et al. 2007). Pellet checks for implicit subclass relationships based on user-defined class relationships. From a development point of view, it also incorporates debugging support for the iterative process of designing and coding a DL error-free ontology. Errors in the ontology have been flagged by error messages, and inconsistent classes are marked for review. Figure 16 shows the result of several verification cycles until the ontology was free of DL errors.

#### 3.6.2. Task-Based Evaluation

This section describes the evaluation in terms of how the ontology can be used to answer questions about certain tasks (France-Mensah and O’Brien 2019), using the RDF query language (SPARQL) for information retrieval to answer sample queries related to the metareasoning problems.

When solving the FTAP problem, the object-level employs three anytime algorithms that are monitored and controlled by a meta-level until a suitable cost- and time-optimized solution is found. In the context of the Allocating Deliberation Time Problem (ADTP), the ontology can answer questions related to the information collected about the several algorithms that can be executed in parallel at the object level. Each algorithm solves one instance of FTAP, but in some circumstances where execution time is limited, the meta-level must select a subset of algorithms to further deliberation. A relevant question in the context of the ADTP is: *Which algorithm was selected by the metalevel to allocate more deliberation time to obtain a better solution that solves the problem at the object level?*

**Figure 17:**
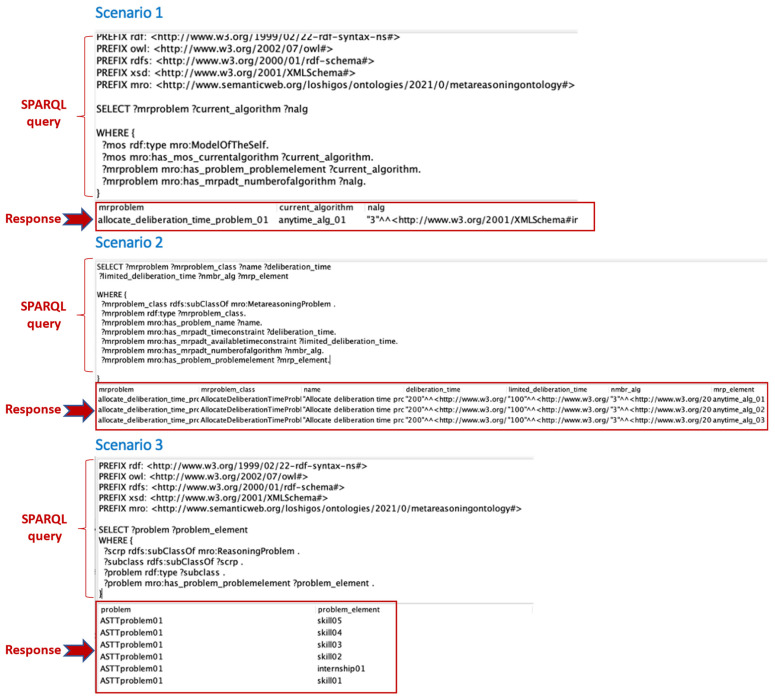
Questions asked to the ontology in SPARQL language.

This question is written in SPARQL query in scenario 1 of Figure 17. In the response, it is observed that the meta-level has selected the anytyme_alg_01, and the ADTP was detected.

The SPARQL query used in the second scenario corresponds to the question: *What is the information related to the configuration of the meta-reasoning problems that the meta-level can solve?* The response of the ontology provides information related to the configuration of the metareasoning problems that the meta-level can solve. In this case, the information is linked between the elements of ADTP metareasoning problem and the current state of the reasoning processes (performance, algorithms, quality of the solution, computational time). This information provides important context for possible actions to take if a metareasoning problem is identified.

In scenario 3, the SPARQL query represents the question: *What are the elements that are part of the configuration of the problem at the object level?* The response of the ontology generates information about the context of the reasoning problems and how to address them.

#### 3.6.3. Data-Driven Evaluation

This section describes the validation process using precision and recall as metrics for evaluating IM-Onto with respect to information retrieval. This evaluation approach makes a comparative analysis consisting of counting the related terms that appear between a predefined set of knowledge elements and the ontology (Guo and Goh 2017). Precision measures the ratio of correctly found knowledge correspondences (true positives) over the total number of returned knowledge correspondences (true positives and false positives) (Brewster et al. 2004). Recall reflects the proportion of knowledge that is accurately detected relative to all the knowledge items it should identify from the predefined set (Brewster et al. 2004). The numerator of Equations (1) and (2) describe that knowledge that is accurately detected and corresponds to the intersection of the relevant entities and the retrieved entities. In each equation, only the denominator differs. As shown in Equation (3), the F-measure we use is F_1, the harmonic means of precision and recall.

(1) $$\mathrm{precision}=\frac{\left|\left\{\mathrm{relevantentities}\right\}\cap \left\{\mathrm{retrievedentities}\right\}\right|}{\left|\left\{\mathrm{retrievedentities}\right\}\right|}$$

(2) $$\mathrm{recall}=\frac{\left|\left\{\mathrm{relevantentities}\right\}\cap \left\{\mathrm{retrievedentities}\right\}\right|}{\left|\left\{\mathrm{relevantentities}\right\}\right|}$$

(3) $$F_{1}=2\times \frac{\mathrm{precision}\times \mathrm{recall}}{\mathrm{precision}+\mathrm{recall}}$$

**Figure 18:**
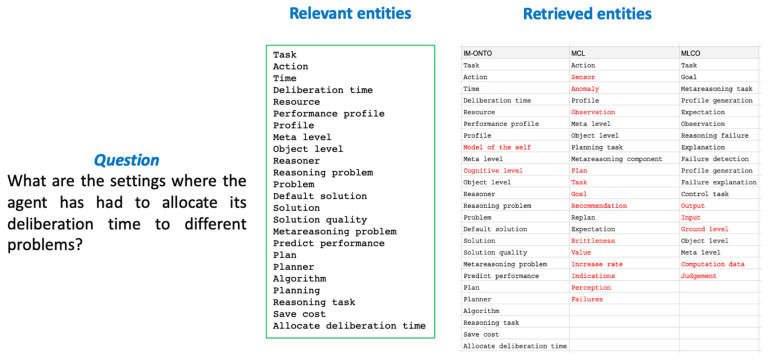
Sample of questions. Manually annotated questions for relevant and retrieved entities. In the response to the question formulated, the terms highlighted in red were not recovered by the corresponding ontologies.

For data-driven evaluation, a corpus in the domain of metareasoning was used. The corpus is a set of answers to questions extracted from five papers that were selected as relevant in the literature review stage but that were not included for the development of the ontology due to the saturation criterion described in Section 3.2.1. For the evaluation, 35 questions were used that covered the information related to the metareasoning problems described in the literature. The questions were manually annotated to extract the main concepts needed to answer them. This is demonstrated in Figure 18. In this figure, a sample question is presented with a list of relevant and retrieved concepts.

Table 5 compares the performance (i.e., recall and precision rates) of IM-Onto with that of both the Metacognitive Loop (MCL) (Schmill et al. 2007, 2011) and Meta-level Control Ontology (MLCO) (Madera-Doval 2019). The precision and recall rate results demonstrate that the low recovery rates of MCL and MLCO reiterate the limitations of existing ontologies and further underscore the need for IM-Onto to support the design of metareasoning systems. On the other hand, the high performance of IM-Onto shows that it contains a high percentage of the relevant entities (recall = 90%) to support the design of systems with integrated support of various types of metareasoning. This reinforces the integrity of the ontology. Similarly, the accuracy rate (91%) of IM-Onto indicates that most of the knowledge elements in the ontology are useful information for the integration of various types of metareasoning in intelligent systems. This supports the conciseness of the ontology.

#### 3.6.4. Criteria-Based Evaluation

For this type of evaluation, the five criteria chosen include competence, consistency, integrity, clarity, and conciseness (El-Diraby 2014). The coherence and consistency criteria were primarily demonstrated by automated consistency checks and task-based assessments, respectively. The data-driven evaluation also demonstrates satisfactory performance in terms of conciseness and completeness through precision analysis and recall, respectively. In the context described, this section focuses on addressing the clarity criterion.

Clarity: The clarity of an ontology is determined when the knowledge elements of the ontology have an unambiguous meaning (El-Diraby 2014). In this study, the terms used for the ontology components were selected from the literature review. The definitions of the terms were obtained by comparing aspects common to all the ontologies studied. In this sense, it is guaranteed that the definitions are objective and independent of the social and computational context.

**Figure 19:**
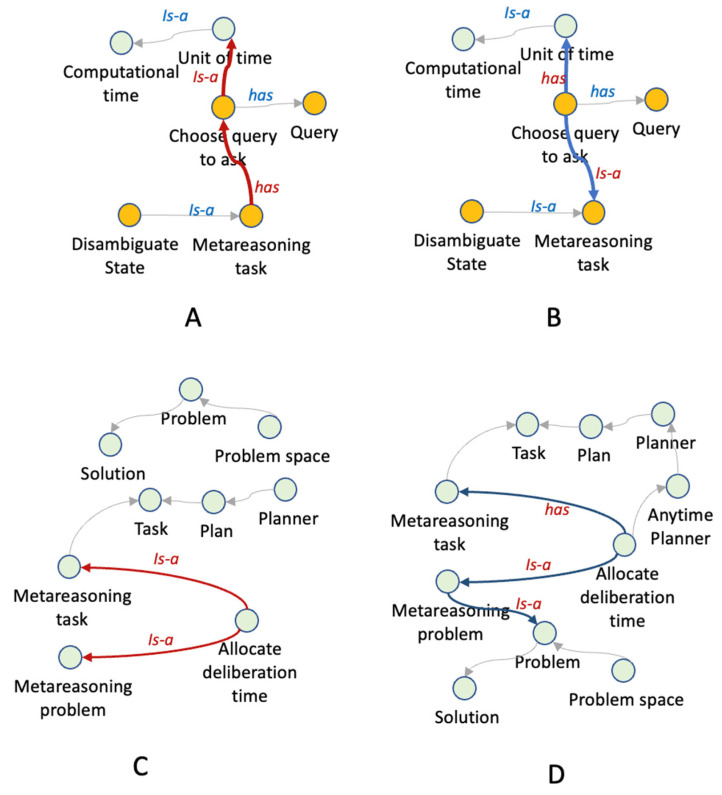
Examples of errors detected in expert validation. Section (**A**) shows two semantic errors in the relations colored in red, and section (**B**) shows the adjustments suggested by the experts. Section (**C**) shows an “incompleteness” error, where the experts commented that class ALLOCATE_DELIBERATION_TIME was inherited from two different classes, which was confusing. Section (**D**) presents the suggestion given by the experts.

Three experts complemented this technical evaluation, where the experts recommended making some adjustments to the ontology due to taxonomic errors that concern the taxonomic structure and are referred to as inconsistency, incompleteness and redundancy. Some examples are presented in Figure 19.

The experts checked those three types of “inconsistency,” both logical and semantic, have been highlighted: circularity errors (e.g., a concept that is a specialization of itself), partitioning errors (e.g., a concept defined as a specialization of two disjoint concepts or a concept defined as a specialization of two different classes), and semantic errors (e.g., a taxonomic relationship in contradiction with the user knowledge). In this sense, the experts found (0) errors of circularity, (1) error of partitioning and (2) semantic errors.

Incompleteness occurs when, for instance, relationships or axioms are missing. In relation to this, the experts found (2) errors. Finally, redundancy occurs when, for instance, a taxonomical relationship can be deduced from others by logical inference. In this topic, the experts found (0) errors.

### 3.7. Ontology Documentation

The IM-Onto ontology itself is available at the following link: https://tinyurl.com/2ymcrk44 (login required). In the documentation, it is possible to find a detailed description of the properties and relationships of each class that is part of the ontology. Additionally included are definitions that allow disambiguating each of the terms that the ontology contains. Similarly, instructions exist at this location for the use of the ontology in information retrieval tasks as well as reasoning tasks for intelligent systems.

## 4. Discussion

The main contribution of this paper was the presentation of a consistent ontology that provides a visual means of sharing a common understanding of the structure and relationships among terms and concepts related to the metareasoning domain in intelligent systems. In this paper, the application of IM-Onto to the problem of allocating student teams to internship programs has been demonstrated in three main tasks. They are (i) providing information linking the elements of a metareasoning problem and the current state of the reasoning processes; (ii) providing information related to the configuration of metareasoning problems the meta-level can solve; and (iii) generating information about the context of the reasoning problems and how to address them.

The high performance of IM-Onto shows that it contains a high percentage of the relevant entities (recall = 90%) to support the design of systems with integrated support of various types of metareasoning. On the other hand, the precision and recall rate results of the validation demonstrate that the low recovery rates of MCL (Schmill et al. 2007, 2011) and MLCO (Madera-Doval 2019) reiterate the limitations of existing ontologies.

The accuracy rate (91%) of IM-Onto indicates that most of the knowledge elements in the ontology are useful information for the integration of various types of metareasoning in intelligent systems. In this sense, MCL and MLCO demonstrate low accuracy rates. These results should be considered when considering the design of intelligent systems with metareasoning capabilities. MCL and MLCO were designed to respond to reasoning failures in intelligent systems, while IM-Onto was designed to handle a wider variety of metareasoning problems.

This study may be expanded in the future to include aspects of metareasoning in humans, metareasoning in inference engines in ontologies and monitoring and control of tasks that expire in time. The inclusion of these three topics can broaden the scope of the IM-Onto ontology, which is currently limited to the domain of metareasoning in intelligent systems. In this sense, it would be interesting to analyze the conceptual differences between metareasoning in human beings, in ontology inference engines and in the monitoring and control of computational processes.

## 5. Conclusions

This paper has presented an ontology called IM-Onto that captures key terms, concepts and relationships related to metareasoning and computational metacognition. The main research objective was to create a common language and conceptualization of metareasoning in the AI domain through the development of an ontology focused on the context of metareasoning problems described in published research. To achieve this, a rigorous research method was followed to guarantee that the two main requirements of the ontology were satisfied (completeness based on relevant knowledge and agreed upon by researchers and practitioners). The research method was based on (Badr et al. 2013), following the phases of definition, conceptualization, formalization, implementation, evaluation, and documentation.

IM-Onto can act as a unifying framework for data sharing across metareasoning problems in an intelligent system. This representation also solves a vocabulary problem in this domain by providing a standard semantic model for cross-functional metareasoning problems. IM-Onto consists of a sub-ontology for each metareasoning problem found on an in-depth analysis of the relevant literature as follows: Allocating Deliberation Time Problem (ADTP), Allocating Evaluation Effort Problem (AEEP), Knowledge Test Problem (KTP), Stopping Reasoning Problem (SRP), Gathering Computational Performance Data problem (GCPDP), Detection of Reasoning Failure Problem (DRFP), Self-explanation and Self-understanding Problem (SE&SUP).

Four approaches were used to evaluate IM-Onto. First, the automated consistency check approach ensures that the ontology is internally consistent. Second, the task-based assessment demonstrated that IM-Onto was able to accomplish the competency tasks defined above using completeness questions. Third, data-driven evaluation rigorously tests the integrity and conciseness of the ontology by demonstrating its comparative performance with previous ontologies. Finally, criteria-based assessment further addresses the ontology clarity metric.

## Figures and Tables

**Table 1:** Literature sources for ontology knowledge and terms used in existing ontologies related to metareasoning.

Literature Source	Ontological Terms
(Schmill et al. 2007, 2011)	sensor, reasoning process, rebuild models, recommendation recover, reinforcement learning, replan, response ontology, result, reward, self-awareness, sensor, sensor failure, sensor malfunction, sensor not reporting, state, system, failure, time, unanticipated perturbation
(Madera-Doval 2019)	agent, metacognition, self-regulation, metamemory, introspective monitoring, meta-level control, cognitive elements, cognitive level, task, reasoning, metareasoning, reasoning task, metareasoning task, object level, cognitive function, perception, situation assessment, categorization, recognition, belief maintenance, problem solving, planning, prediction, expectation, sensor, observation

**Table 2:** Papers reviewed for the stage of gathering information, knowledge acquisition and conceptualization.

Research Paper	Terms per Paper	Citations per Paper *
(Russell and Wefald 1991)	48	444
(Conitzer and Sandholm 2003)	67	41
(Cox 2005)	63	261
(Anderson and Oates 2007)	36	91
(Schmill et al. 2007)	77	21
(Cox and Raja 2008)	55	100
(Chen et al. 2013)	37	3
(Lin et al. 2015)	54	46
(Lieder and Griffiths 2017)	37	23
(Ackerman and Thompson 2017)	36	105
(Milli et al. 2017)	35	39
(Cserna et al. 2017)	34	9
(Karpas et al. 2018)	22	5
(Farmer 2018)	7	5
(Madera-Doval 2019)	27	1
(Houeland and Aamodt 2018)	25	5
(Parashar et al. 2018)	33	1
(Griffiths et al. 2019)	18	29
(Svegliato et al. 2018)	41	16
(Sung et al. 2021)	36	0

* Google Scholar was the source for the number of citations of the papers at the time of writing this article. Data collected in June 2021.

**Table 3:** List of terms used for class definition in the ontology.

Classes of the Ontology
action, action selection, agent, algorithm, allocate deliberation time, allocating evaluation effort, answer, anytime algorithm, anytime planner, assess anomaly, bayes algorithm, best action, calculate quality of current solution, choose query to ask, cognitive level, cognitive problem, cognitive task, component, computational method, computational step, computational step result, computational time, compute expected utility, compute performance projection, compute solution quality, constraint, control, control policy, current state of the world, default action, default solution, disambiguate state, evaluation task, evaluation test, event, expectation, explain failure, explanation, failure, failure explanation, failure state, function, generate expectation, generate learning goals, get current solution, goal, ground level, ground-level story, guided response, imxp, increment time step, information gathering, information gathering policy, information gathering task, initialize performance history, initialize time step, internal state, introspection, itmxp, knowing current state of the world, knowledge test, learner, learning goal, limited time, make recommendation, mental action, meta level, metacognitive loop, metacognitive problem, metalevel control, metareasoner, metareasoning component, metareasoning problem, metareasoning task, model, model of the self, model of the word, monitor behavior, monotonicity, nag cycle, neural network, nonlinear regression, note anomaly object level, object-level process observation, online control policy, optimal information gathering policy, optimal test policy, outcome state, perception, performance history, performance predictor, performance profile, performance projection, perturbation, perturbation detection, phase, plan, planner, planning, planning task, policy, predict performance, problem, process, profile, problem space, property, question, recommendation action, reactivate learning, reasoner, reasoning, reasoning failure, reasoning problem, reasoning strategy, reasoning system, reasoning task, recommendation, recover, replan, resource, result, run test, save costs, selecting action, sensor, situation assessment, sleep, solution, solution quality, start acting, start anytime algorithm, state, state of the world, stop reasoning, stopping condition, story, story understanding task, strategy, success state, system, task, test, test policy, time, time dependent utility function, trace, unanticipated perturbation, understanding task, unit of time, unusual event, utility function, vector, violated expectation

**Table 4:** Properties and domain range of two classes in the ontology.

Class	Property	Domain	Value Restriction
MetaLevel	rdf:subClassOf	CognitiveLevel	
	has_problemspace	ProblemSpace	Non-empty array
	has_metareasoner	Metareasoner	An algorithm
	has_current_metareasoning_loop	Integer	Positive integer
MetareasoningTask	rdf:subClassOf	MetacognitiveTask	
	has_goal	Goal	
	has_id	String	Unique value
	has_input	Array	An array of objects
	has_output	Array	Non-empty
	has_runtime	Number	Positive number
	has_preconditions	State	Non-empty array of states
	has_effects	State	Non-empty array of states
	has_name	String	Alphanumerical value

**Table 5:** Precision and recall rates for comparing ontologies.

Ontologies	Precision	Recall	F-Measure
IM-Onto	92%	90%	91%
MCL (Schmill et al. 2007, 2011)	47%	37%	41%
MLCO (Madera-Doval 2019)	63%	45%	53%

## Data Availability

The data presented in this study are available on request from the corresponding author. The data are not publicly available due to privacy issues and property rights. The IM-Onto ontology is available at the following link (account creation and login required). https://tinyurl.com/2ymcrk44.
